# Molecular imaging of inflammation with PET in acute and ventilator-induced lung injury

**DOI:** 10.3389/fphys.2023.1177717

**Published:** 2023-06-29

**Authors:** Guido Musch

**Affiliations:** Department of Anesthesiology and Perioperative Medicine, UMass Chan Medical School, Worcester, MA, United States

**Keywords:** inflammation, positron-emission tomography, ventilator-induced lung injury, acute lung injury, respiratory distress syndrome, isotopes

## Abstract

This review focuses on methods to image acute lung inflammation with Positron Emission Tomography (PET). Four approaches are discussed that differ for biologic function of the PET reporter probe, radiotracer employed, and the specific aspect of the inflammatory response that is targeted. 2-[^18^F]fluoro-2-deoxy-D-glucose ([^18^F]FDG) is an enzyme substrate whose uptake is used to measure the metabolic activation of inflammatory cells during acute lung injury in the noncancerous lung. H_2_^15^O and radiolabeled plasma proteins are inert molecules with the same physical characteristics as their nonradioactive counterparts and are used to measure edema and vascular permeability. Tagged enzyme or receptor inhibitors are used to probe expression of these targets induced by inflammatory stimuli. Lastly, cell-specific tracers are being developed to differentiate the cell types that contribute to the inflammatory response. Taken together, these methods cast PET imaging as a versatile and quantitative tool to measure inflammation *in vivo* noninvasively during acute and ventilator-induced lung injury.

## 1 Introduction

Pulmonary inflammation is a hallmark of acute lung injury and its form induced by mechanical ventilation (ventilator-induced lung injury, VILI). Because this inflammation is considered a driver of outcome and hence a target of therapeutic approaches, significant interest has developed around methods to detect, measure, and monitor it *in vivo* noninvasively in the lung as the inflammatory process develops. In this respect, Positron Emission Tomography (PET) has emerged as one of the favorite imaging methods because of its versatility. The possibility to label molecules that participate in the biochemical reactions of inflammatory processes with positron emitters, and follow their fate *in vivo*, noninvasively, and with 3-dimensional resolution without altering their chemical properties has led to an array of PET techniques that target various pathways of the inflammatory process. Not aiming to be an exhaustive review of this topic, the emphasis herein will be on examples of PET imaging methods that leverage different aspects of the inflammatory response.

## 2 Imaging inflammatory cell metabolic activity with 2-[^18^F]fluoro-2-deoxy-D-glucose ([^18^F]FDG)

Substantive experimental and clinical evidence has established PET measurement of [^18^F]FDG uptake as a method to noninvasively assess activation of inflammatory cells, in particular neutrophils, in the noncancerous lung (Jones et al., 1994; Jones et al., 1997; Chen and Schuster, 2004). In this section, the molecular mechanisms underlying the [^18^F]FDG signal, and its biologic significance, will be discussed first, followed by findings of studies that employed [^18^F]FDG to gain insights into the pathophysiology of the acute respiratory distress syndrome (ARDS) and VILI and on the effect of interventions to ameliorate these conditions.

Cells activated by an inflammatory stimulus increase their glucose consumption to satisfy the ensuing energy requirement. Neutrophils do so to an exaggerated extent because they possess few mitochondria and rely primarily on glycolysis to sustain their functional responses (Bao et al., 2014). The low ATP yield of glycolysis implies that activated neutrophils consume much higher quantities of glucose than other cell types. This phenotypic trait can be leveraged by administering the positron emitting glucose analog [^18^F]FDG, which is taken up by metabolically active cells through the facilitative glucose transporters, particularly Glut-1 and Glut-3 (Wang et al., 2016), and phosphorylated by hexokinase. Studies have shown that deoxyglucose uptake is related to plasma membrane levels of Glut-1 and to hexokinase activity (Haberkorn et al., 1994; Torizuka et al., 1995; Paik et al., 2005a; Paik et al., 2005b; Schuster et al., 2007). [^18^F]FDG is thus an ideal tracer to measure neutrophil activation because it undergoes the two rate limiting steps of glycolytic metabolism, i.e., transport and phosphorylation (Torizuka et al., 1995; Paik et al., 2005b), on which the energy requirement for all of the activated neutrophil’s functions rests. [^18^F]FDG-6-phosphate, however, cannot proceed further along the glycolytic pathway nor leave the cell. It accumulates intracellularly in proportion to metabolic rate, yielding a signal that can be imaged by PET. Glut-1 translocation from the cytoplasm to the cell surface (Schuster et al., 2007) and increased transporter affinity (Tan et al., 1998) are the main mechanisms of increased deoxyglucose transport into activated neutrophils.

Tracer kinetic models have been developed to measure the rates of glucose transport and phosphorylation with PET (Phelps et al., 1979). Because some assumptions of those models may not be valid in the acutely injured lung, we developed a tracer kinetic model specific for acute lung injury that accounts for [^18^F]FDG distribution in edematous tissue (Schroeder et al., 2008). This model allows estimation of the extravascular extracellular volume of distribution of [^18^F]FDG and of the rates of glucose transport (k_1_) and phosphorylation (k_3_) in acutely injured lungs. Consequently, this model allows noninvasive quantitative assessment of the key molecular steps that control cellular utilization of glucose in a subject with acute lung injury. Using this model we showed that, under conditions of surfactant depletion and endotoxemia, two common causes of acute lung injury, [^18^F]FDG PET-derived parameters correlated with biologically relevant effectors of inflammation, as the [^18^F]FDG phosphorylation rate paralleled expression of IL-1β, IL-8, and IL-10, all cytokines implicated in VILI and acute lung injury, and the [^18^F]FDG volume of distribution increased with the amount of infiltrating neutrophils. (de Prost et al., 2014).

The cellular events and signaling pathways responsible for increased glucose transport and phosphorylation in the activated neutrophil have been investigated *in vitro*. Such studies have shown that the increase in deoxyglucose uptake occurs during leukocyte priming (Tan et al., 1998; Jones et al., 2002a; Paik et al., 2004), for instance by TNF-α (Jones et al., 2002a). This finding supports the use of [^18^F]FDG to measure neutrophil activation because priming is the first step in neutrophil activation and is critical for neutrophil-mediated tissue injury. Studies *in vitro* have shown that the molecular pathway for increased [^18^F]FDG uptake involves several protein kinases, such as phosphatidylinositol-3 kinase (Paik et al., 2005a), tyrosine kinase, and protein kinase C (Tan et al., 1998; Paik et al., 2004), which affect the expression or activity of glucose transporters and hexokinase and are implicated in neutrophil priming.

In a unilateral model of VILI, we used PET of [^18^F]FDG to demonstrate that tidal overdistension and end-expiratory alveolar derecruitment are accompanied by increased [^18^F]FDG uptake (Musch et al., 2007), which could be significantly reduced by application of positive end-expiratory pressure (PEEP). The increase in [^18^F]FDG uptake during VILI is largely attributable to neutrophils and, to a lesser extent, other cell populations such as macrophages and type 2 epithelial cells (Musch et al., 2007; Saha et al., 2013). Subsequent studies have employed this technique to demonstrate that, in VILI, the spatial distribution of [^18^F]FDG uptake is related to that of lung mechanical strain (Retamal et al., 2018) and that an increase in regional pulmonary [^18^F]FDG uptake is accompanied by overexpression of genes implicated in the pathogenesis of VILI, such as genes that encode for epithelial and endothelial stretch markers and genes involved in specific inflammatory pathways (Wellman et al., 2016; Motta-Ribeiro et al., 2018). The topographical heterogeneity of pulmonary [^18^F]FDG uptake in VILI is enhanced by infusion of low-dose endotoxin during mechanical ventilation (Costa et al., 2010), a model for clinical sepsis, and reduced by protective ventilation with high PEEP and low tidal volume (de Prost et al., 2013). Specifically, endotoxin is synergistic with mechanical strain as lipopolysaccharide infusion amplified the effect of tidal strain on [^18^F]FDG phosphorylation rate 3-fold (Wellman et al., 2014). This is consistent with a priming effect of endotoxin on the mechanically ventilated lung, acting as a “first hit” that renders the lung more vulnerable to the inflammatory effect of mechanical ventilation.

Inflammation imaging with PET of [^18^F]FDG uptake has provided important insights also into the pathophysiology of acute lung injury due to causes other than mechanical ventilation, and of ARDS. In a sheep model of acute cotton smoke inhalation, we demonstrated that inflammatory cell metabolic activation occurs early and precedes impairment of pulmonary gas exchange (Musch et al., 2014). This finding is potentially clinically important because it implies that the therapeutic window for antiinflammatory therapies and application of protective mechanical ventilation might be soon after smoke inhalation and before the clinical picture of ARDS ensues. Chen et al. (Chen and Schuster, 2004) showed that when the acute injury was mainly characterized by alteration of pulmonary vascular permeability and edema, as in the oleic acid model, the increase of [^18^F]FDG uptake was minor and not statistically significant. In contrast, the rate of [^18^F]FDG uptake was significantly elevated after a low dose infusion of endotoxin, both with and without concomitant oleic acid injury, even in the absence of alveolar neutrophilia. This finding is important because it established the specificity of [^18^F]FDG PET for measuring neutrophil activity in acute lung injury.

**FIGURE 1:**
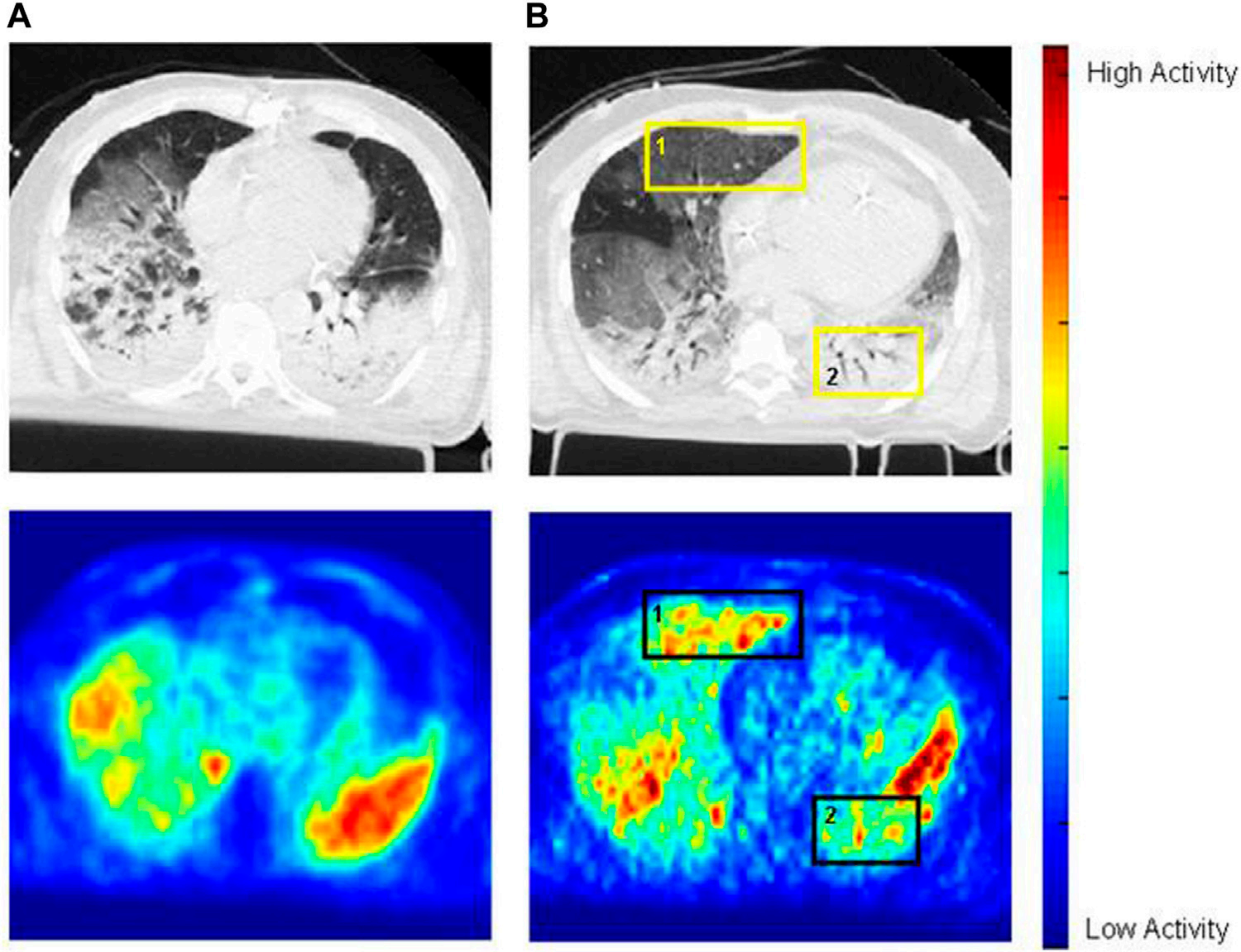
Images of cross-registered CT (top row) and [^18^F]FDG PET (bottom row) in two mechanically ventilated patients with ARDS. In patient A (images on the left), [^18^F]FDG distribution parallels that of the opacities on CT. In patient B (images on the right), [^18^F]FDG uptake is higher in normally aerated regions than in dorsal opacified regions (e.g., square 1 *versus* square 2, or ventral half of the left lung *versus* its dorsal half–square 2). Reprinted from Bellani et al. (2009), with permission.

[^18^F]FDG PET imaging has been applied also to patients with ARDS. An early case report showed markedly increased and diffuse pulmonary [^18^F]FDG uptake in a patient with ARDS (Jacene et al., 2004). The authors hypothesized that high rates of glucose utilization by the inflammatory cells involved in ARDS pathogenesis were responsible for the [^18^F]FDG signal. A later study confirmed this finding in a larger cohort of patients and further showed that [^18^F]FDG uptake was higher than in control subjects even in lung regions with normal density on computed tomography (Bellani et al., 2009). This finding is important because it implies that, in ARDS, also lung regions that appear normal radiographically are inflamed and, consequently, the functionally defined “baby lung” is not necessarily a healthy lung. A subsequent analysis shed further light on the cause of the elevated [^18^F]FDG uptake in normally aerated lung regions of ARDS patients by reporting a direct correlation between [^18^F]FDG uptake of these regions and their tidal mechanical strain (Bellani et al., 2011). This finding suggests that the inflammation of the baby lung is, at least in part, related to the strain imposed by mechanical ventilation, i.e., to VILI. This ability to topographically correlate the [^18^F]FDG signal with regional density and mechanics, afforded by combined PET/CT imaging, can help differentiate when the inflammatory cell metabolic activation is due mainly to the primary insult causing ARDS or to the secondary insult from mechanical ventilation, even though neutrophil activation is a hallmark of both these processes. In this respect, Bellani et al. (Bellani et al., 2009) described distinct patterns of [^18^F]FDG uptake in patients with ARDS. In some patients, uptake was highest in regions classified as nonaerated on CT, which are likely those most affected by the primary inflammatory process causing ARDS. In these patients, it is reasonable to assume that the [^18^F]FDG signal primarily reflected neutrophil activation due to the primary insult. In other patients, instead, the [^18^F]FDG uptake rate was higher in normally or mildly hypo aerated regions, suggesting that inflammatory cell activation in these patients could have been mainly due to the secondary insult from mechanical ventilation, as these are the regions distended by the tidal volume or that potentially undergo injurious cyclic tidal recruitment and derecruitment (Figure 1).

## 3 Imaging edema and pulmonary vascular permeability

A hallmark of any form of acute lung injury, including VILI and ARDS, is increased pulmonary vascular permeability, leading to interstitial and alveolar edema. One of the first applications of PET to functional lung imaging assessed these pathophysiological processes. That application relies on oxygen-15 labeled water (H_2_^15^O) as the radiotracer. H_2_^15^O has the same physical properties as water. In particular, it diffuses freely across the pulmonary endothelium, so that the tracer rapidly reaches equilibrium with lung tissue. Regional lung water can be calculated by referencing lung tissue activity at equilibrium, measured with PET, to the activity of blood water, measured from blood samples collected during the scan. Regional lung water includes both intravascular (blood) and extravascular lung water. Because the pathophysiological variable of interest is edema, an additional measurement is necessary to parse out extravascular lung water. Intravascular lung water is computed by taking a PET scan after administration of a blood pool compartment label such as carbon monoxide, which binds to hemoglobin with high affinity and can be tagged with positron emitters carbon-11 (^11^C) or oxygen-15 (^15^O). Extravascular lung water is then obtained by subtracting intravascular water, measured with an inhalation PET scan of ^11^CO or C^15^O, from regional water measured with an intravenous infusion scan of H_2_^15^O (Schuster et al., 1985; Schuster, 1989).

In acute and ventilator-induced lung injury, edema is a consequence of increased pulmonary vascular permeability. The possibility to label proteins that cross the alveolo-capillary membrane only in pathologic conditions of increased permeability with positron emitters enables PET to detect and measure this pathophysiologic alteration. A specific measure of pulmonary vascular permeability can thus be derived by measuring with PET the transport rate constant(s) (pulmonary transcapillary escape rate, PTCER) of a radiolabeled protein, such as ^68^Ga-transferrin or ^11^C-methylalbumin, between the intravascular and extravascular space (Schuster, 1989; Schuster et al., 1998).

Using these techniques, Sandiford et al. (Sandiford et al., 1995) reported that pulmonary vascular permeability, measured in ARDS patients *in vivo* from the PTCER of ^68^Ga-transferrin, was higher than in normal subjects and, within the ARDS group, they did not find consistent differences in PTCER between ventral and dorsal lung regions. In contrast, they reported that extravascular lung water was significantly higher in dorsal, dependent regions, than in ventral, nondependent ones. These data are consistent with the subsequent findings of the [^18^F]FDG studies mentioned above and further point to the fact that the functionally defined “baby lung” is not a healthy, normal lung. Instead, the lung in ARDS is globally inflamed even if the resulting increase in lung density may be heterogeneously distributed and edema predominantly dependent because of the effect of gravity.

## 4 Imaging enzymes and receptors involved in lung inflammation

Recently, PET techniques have been developed to image specific molecular targets involved in inflammation. Inducible nitric oxide synthase (iNOS) is expressed in pulmonary epithelium, increased by pulmonary inflammation, and associated with disease severity in ARDS. Because of the central role of iNOS in inflammation, efforts have been made to develop iNOS inhibitors as pharmaceutical agents, and radiolabeled iNOS inhibitors for probing iNOS expression *in vivo* using PET. One such radiotracer, named ^18^F-NOS, was tested to detect pulmonary iNOS expression in murine models after intravenous or intratracheal endotoxin administration (Zhou et al., 2009). In that study (Zhou et al., 2009), there was a progressively higher *relative* uptake of ^18^F-NOS in the lungs of mice pretreated with i.v. endotoxin compared with untreated controls up to 1 hour after administration of the tracer but this differential uptake almost entirely waned by 2 hours, pointing to the ideal kinetics of this tracer to image pulmonary inflammation. These biodistribution results paralleled the pulmonary levels of iNOS assessed by Western blot in the same mice, consistent with iNOS-specific uptake of ^18^F-NOS, a conclusion confirmed by iNOS blocking studies. MicroPET images obtained over 1 hour in mice who had received intratracheal endotoxin confirmed higher pulmonary uptake of ^18^F-NOS than in control mice. These animal studies were corroborated by a recent study in humans that showed a ∼30% increase in ^18^F-NOS uptake with PET 16 h after instillation of endotoxin in the right middle bronchus (Huang et al., 2015). This increased uptake was topographically associated with an increase in density on computed tomography of the area of endotoxin instillation and with positivity for iNOS of bronchoalveolar lavage cells recovered from that area and stained immunohistochemically. Taken together, these studies demonstrated the potential of ^18^F-NOS to image iNOS activity in acute lung inflammation *in vivo*, including in humans.

Peroxisome proliferator-activated receptor gamma (PPARγ) has a role in metabolism and inflammation, and its agonists have been investigated in the treatment of a variety of diseases. PET ligands that bind to PPARγ have been developed to monitor receptor expression over time and potentially identify patients who would most likely benefit from PPARγ directed therapies (Lee et al., 2012).

## 5 PET imaging of lung macrophages

Although primed and activated neutrophils are the main source of the [^18^F]FDG signal, other cell types also contribute to the uptake of [^18^F]FDG. Specifically in relation to acute lung inflammation, Saha et al. (Saha et al., 2013) demonstrated that alveolar macrophages give an appreciable contribution to the [^18^F]FDG signal both in VILI and in a model of acute lung injury caused by intranasal instillation of endotoxin. Consequently, there is considerable interest in PET tracers that can selectively tag macrophages. A class of these tracers is represented by translocator protein (TSPO) ligands. TSPO is a transmembrane mitochondrial channel overexpressed in macrophages and monocytes in various inflammatory states. Jones et al. (Jones et al., 2002b) showed that PET imaging of the TSPO ligand PK11195 labeled with ^11^C revealed macrophage accumulation and trafficking in a rabbit model of lung injury induced by endobronchial instillation of silica particles. Radioactive counts from the challenged right upper lobe were elevated compared to control lung regions, and paralleled macrophage accumulation. With time, the signal increased also in regions along the lymphatic drainage path, consistent with macrophage trafficking through lymph ducts. In contrast, neither radioactivity nor macrophage numbers increased in lung regions challenged with endobronchial *S. pneumoniae*, despite marked neutrophilia on histology and demonstration in a prior study (Jones et al., 1994) that the *S. pneumoniae* model was associated with markedly increased [^18^F]FDG uptake. A subsequent study in humans by the same group (Jones et al., 2003) corroborated the concept that ^11^C- PK11195 and [^18^F]FDG mostly target two different cell types, i.e., macrophages and neutrophils, respectively, which are involved to a different extent in asthma *versus* chronic obstructive pulmonary disease (COPD).

More recently, Chen et al. (Chen et al., 2021) reported on a newer TSPO PET ligand with greater affinity for TSPO and specificity for lung macrophages, N-acetyl-N-(2-[^11^C]methoxybenzyl)-2-phenoxy-5-pyridinamine, which also can be labeled with ^11^C ([^11^C]PBR28). [^11^C]PBR28 uptake increased concomitantly with the development of a M2 macrophage-dominant lung inflammatory response in a Sendai virus infection model. In contrast, [^11^C]PBR28 uptake did not rise in an endotoxin instillation model characterized by neutrophilic inflammation. This differential uptake of [^11^C]PBR28 between the two models contrasted with that of [^18^F]FDG, which increased similarly in both. The increase in [^11^C]PBR28 uptake, but not [^18^F]FDG, was attenuated in transgenic mice heterozygous for a mutation that confers macrophage depletion (*Csf1* ^*wt/opT*^). Lung tissue immunohistochemical staining revealed that TSPO localized predominantly to macrophages rather than neutrophils.

Taken together, these results suggest that TSPO ligands, especially those of the later generation with higher affinity, may yield useful PET tracers to assess the pulmonary macrophage inflammatory response and complement the less specific metabolic information provided by [^18^F]FDG.

## 6 Conclusion

Over the past several years, substantial advancements in PET technology, radiochemistry, and molecular biology have enabled development of an array of imaging methods to study inflammation in the acutely injured lung. These methods have yielded fundamental insights into the pathophysiology of VILI and ARDS.
